# Stoichiometry, polarity, and organometallics in solid-phase extracted dissolved organic matter of the Elbe-Weser estuary

**DOI:** 10.1371/journal.pone.0203260

**Published:** 2018-09-05

**Authors:** Kerstin B. Ksionzek, Jing Zhang, Kai-Uwe Ludwichowski, Dorothee Wilhelms-Dick, Scarlett Trimborn, Thomas Jendrossek, Gerhard Kattner, Boris P. Koch

**Affiliations:** 1 Alfred Wegener Institute Helmholtz Center for Polar and Marine Research, Bremerhaven, Germany; 2 MARUM–Center for Marine Environmental Sciences, Leobener Straße, Bremen, Germany; 3 State Key Laboratory of Estuarine and Coastal Research (SKLEC), East China Normal University (ECNU), Shanghai, China; 4 University of Applied Sciences, Bremerhaven, Germany; National Sun Yat-sen University, TAIWAN

## Abstract

Dissolved organic matter (DOM) is ubiquitous in natural waters and plays a central role in the biogeochemistry in riverine, estuarine and marine environments. This study quantifies and characterizes solid-phase extractable DOM and trace element complexation at different salinities in the Weser and Elbe River, northern Germany, and the North Sea. Dissolved organic carbon (DOC), total dissolved nitrogen (TDN), Co and Cu concentrations were analyzed in original water samples. Solid-phase extracted (SPE) water samples were analyzed for DOC (DOC_SPE_), dissolved organic nitrogen (DON_SPE_), sulfur (DOS_SPE_) and trace metal (^51^V, ^52^Cr, ^59^Co, ^60^Ni, ^63^Cu, ^75^As) concentrations. Additionally, different pre-treatment conditions (acidification vs. non-acidification prior to SPE) were tested. In agreement with previous studies, acidification led to generally higher recoveries for DOM and trace metals. Overall, higher DOM and trace metal concentrations and subsequently higher complexation of trace metals with carbon and sulfur-containing organic complexes were found in riverine compared to marine samples. With increasing salinity, the concentrations of DOM decreased due to estuarine mixing. However, the slightly lower relative decrease of both, DOC_SPE_ and DON_SPE_ (~77%) compared to DOS_SPE_ (~86%) suggests slightly faster removal processes for DOS_SPE_. A similar distribution of trace metal and carbon and sulfur containing DOM concentrations with salinity indicates complexation of trace metals with organic ligands. This is further supported by an increase in Co and Cu concentration after oxidation of organic complexes by UV treatment. Additionally, the complexation of metals with organic ligands (analyzed by comparing metal/DOC_SPE_ and metal/DOS_SPE_ ratios) decreased in the order Cu > As > Ni > Cr > Co and thus followed the Irving-Williams order. Differences in riverine and marine trace metal containing DOM_SPE_ are summarized by their average molar ratios of (C_107_N_4_P_0.013_S_1_)_1000_V_0.05_Cr_0.33_Co_0.19_Ni_0.39_Cu_3.41_As_0.47_ in the riverine endmember and (C_163_N_7_P_0.055_S_1_)_1000_V_0.05_Cr_0.47_Co_0.16_Ni_0.07_Cu_4.05_As_0.58_ in the marine endmember.

## Introduction

Dissolved organic matter (DOM) is actively cycling in natural waters and participates in most biogeochemical processes. Assessment of the DOM stoichiometry supports to unravel its origin and fate and to understand its role in different aquatic environments. The biogeochemistry of marine dissolved organic carbon, nitrogen and phosphorus (DOC/N/P) was extensively studied in the past, e.g. [1, 2]. In contrast, the knowledge on quantity, distribution, and the biogeochemical role of dissolved organic sulfur (DOS) in aquatic environments is limited, though not less important. We have previously estimated the global marine DOS inventory to range between 6.7 and 18.6 Pg S [3]. In particular, rivers are known to be important sources of reduced carbon, nitrogen and phosphorus to coastal environments [4]. Riverine DOM can be influenced by different transformation and removal processes along its way into estuarine and marine water: salt-induced flocculation [5, 6], adsorption to particulate matter [7, 8], photo-oxidative remineralization [9], and uptake by heterotrophs [10, 11]. Thus, typical concentrations of DOC and DON decrease over a salinity gradient from riverine to marine water [12, 13]. DOC/DON ratios usually also decrease from land to sea along the salinity gradient in estuaries [14] indicating differences in the stoichiometry of the organic matter precursors. In our previous study, we used existing literature and roughly estimated that the riverine transport of organic sulfur in particulate (POS) and dissolved form combined is about 0.25 Tmol S a^-1^ (8 Tg S a^-1^) [3]. In estuarine and marine environments, the concentration of sulfate (up to 29 mmol S L^-1^) exceeds the concentration of DOS by up to five orders of magnitude. As the analysis of DOS has been analytically hampered, the composition and biogeochemistry of DOS remains widely unknown. Several studies focused primarily on volatile organic sulfur compounds, such as dimethylsulfide (DMS) and carbonyl sulfide (COS), because they are actively involved in climate processes [15–17]. However, those climate relevant organic sulfur compounds contribute less than 3% to the total marine DOS pool [3].

Other organic sulfur compounds, such as sulfides and thiols, play an important role as ligands for organic metal complexes [18]. Thiols build strong complexes with copper and account for a major part of the copper complexing ligand pool in surface seawater [19, 20]. Silver and mercury are also known to bind strongly with organic sulfur species [21, 22]. Organic metal-complexing ligands can thus affect the mobility, toxicity, and bioavailability of several trace metals. Some metals or metalloids in aquatic ecosystems, such as As, Co, Cu, Cr, Ni, and V are essential micronutrients to support biological processes [23–26], e.g Cu, Co, and Ni are essential for growth and control of marine phytoplankton populations [27]. This has also an indirect effect on bioproductivity, species composition and, in the long term, food web dynamics. However, in high concentrations, these metals can also cause toxic effects [26, 28]. Reduced toxicity was found for some trace metals (e.g. Cu, Pb, Cd) in case of higher DOM complexation rates [29, 30]. Moreover, trace metals can not only trigger the active production of organic ligands but also contribute to their persistence in surface waters: trace-metal complexation has a protective effect against oxidation of DOM-thiol groups [31], whereas the production of copper-binding thiols is enhanced with increasing copper-levels [32–35]. Besides quantity, the quality of DOM plays also an equally important role for trace metal complexation [36]. Baken et al. found that increasing aromaticity lead to a higher trace metal affinity of DOM, indicating that aromatic humic substances can act as major metal chelators [36]. Matar et al. [37] analyzed the influence of organic matter polarity on trace metal speciation and bioavailability and revealed that the hydrophobic DOM fraction has a lower binding capacity for Cu than the hydrophilic fraction, suggesting lowered Cu bioavailability in presence of hydrophilic DOM. Although DOM interactions with metals and the distribution and cycling of organic metal complexes are a growing field of interest, the influence of DOM and specifically of DOS compounds on transport, kinetics, bioavailability and toxicity of trace metals remains largely unknown.

Here we present results on the composition and distribution of DOM at different salinities sampled from the rivers Weser and Elbe in northern Germany to the marine waters of the North Sea. Our aim is to improve our knowledge on distribution and composition of organically bound trace metals. The major research questions/hypotheses are:

- What is the concentration of solid-phase extractable DOS (DOS_SPE_) in the rivers Elbe and Weser and how does it change with salinity? How does the stoichiometry (molar elemental ratios) of solid-phase extractable DOM (DOM_SPE_) change with salinity within the Elbe-Weser-Estuary?
- Since some sulfur species, such as thiols, are known as trace-metal ligands, we hypothesize that the amount of organically-bound trace metals correlates with the relative contribution of DOS and DOM. Consequently, changes in DOM concentration with salinity should also be reflected in trace metal concentrations.
- How does DOM polarity change along the estuary and is this change connected to dissolved/complexed trace element concentrations?
- What is the influence of salinity and different sample pre-treatment conditions (pH 2 versuss pH 8 extraction) on DOM and associated trace element composition?

## Materials and methods

### Sample collection and processing

**Fig 1:**
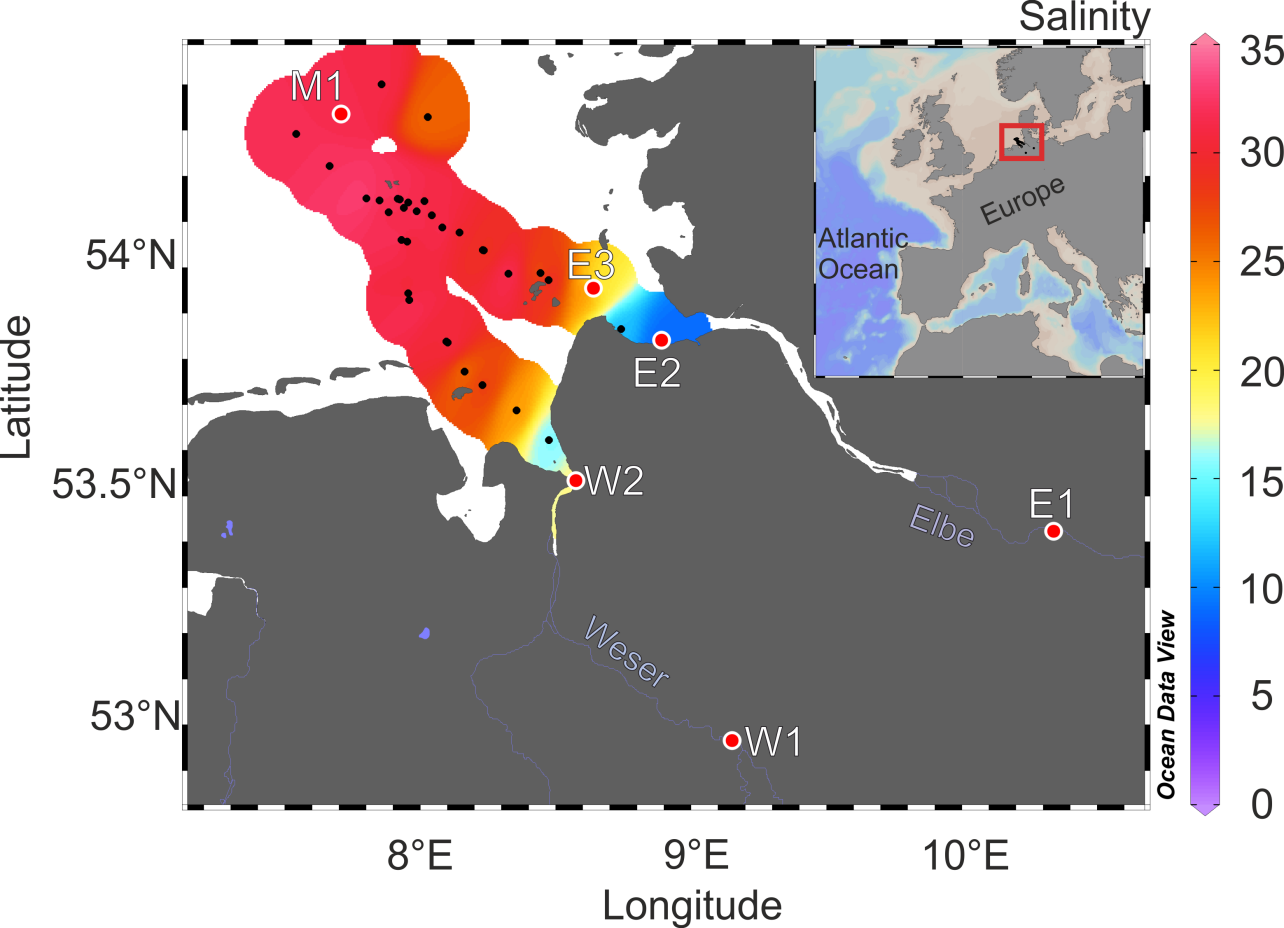
Map of sampling area. Sampling locations are marked as red dots, colors represent the surface salinity. Black dots represent stations, at which temperature and salinity were measured [39].

**Fig 2:**
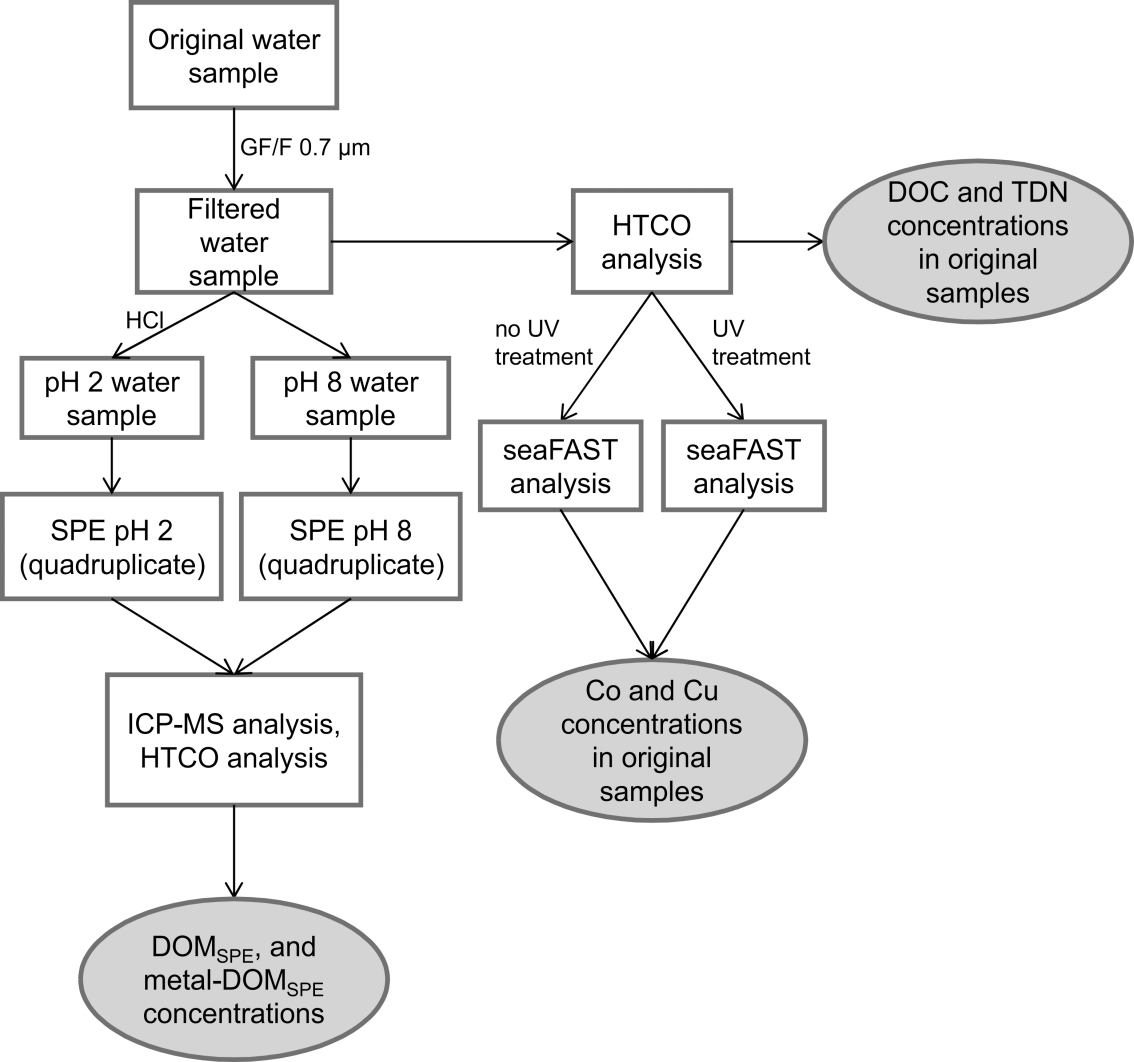
Sample processing workflow. The sample processing steps are represented as white boxes. Measured parameters are specified in grey boxes. DOC and total dissolved nitrogen (TDN) in original water samples were analyzed by high temperature catalytic oxidation (HTCO). The seaFAST analysis was used to determine Co and Cu concentrations in the original filtered water samples. Aliquots of original filtered water samples were solid-phase extracted in quadruplicates for each acidified and non-acidified sample. DOC and DON in solid-phase extracts (DOC_SPE_, DON_SPE_) were analyzed by HTCO. All other elements were analyzed by inductively coupled plasma mass spectrometry (ICP-MS).

Six surface water samples were collected in June and July 2014 from Rivers Weser and Elbe, northern Germany (salinity ~ 0), and in the Southern North Sea (salinity ~33, Fig 1, Table 1). The marine water sample (M1) and samples from the Elbe Estuary (E2, E3) were collected with a rosette sampler connected to a conductivity, temperature, and depth sensor (CTD) (expedition HE426 of *R/V Heincke*). In total, 36 CTD stations were performed to analyze background parameters, such as temperature and salinity. Other riverine samples from River Elbe (E1) and River Weser (W1, W2) were collected manually in glass bottles. Temperature, conductivity, and pH were measured in situ with a sensor (Cond 340i, WTW). No specific permissions were required for sampling and the field studies did not involve endangered or protected species. The sample processing workflow is presented in Fig 2. All samples were filtered through pre-combusted GF/F filters (Whatman, 450°C, 5 h, 0.7 μm nominal pore size) with a maximum pressure < 200 mbar. Aliquots for DOC and nutrient analyses were stored at -20°C in pre-cleaned high-density polyethylene (HDPE) bottles. Filtered water was either acidified to pH 2 (hydrochloric acid, suprapur, Merck) or processed without acidification (pH ~8). SPE was applied for DOM enrichment and desalting [38] and for each sample 500 mL of filtered water (pH 8 and pH 2) was extracted (PPL, 200 mg, Mega Bond Elut, Varian) in quadruplicates and each eluted with ~1 mL methanol (LiChrosolv, Merck; exact volume was determined by weighing) into pre-combusted glass vials. After extraction, DOM_SPE_ was stored at -20°C until further analysis.

**Table 1:** Sampling locations and hydrographic conditions.

Sample	Location	Date	Salinity	Temperature (°C)	Category
W1	52.965°N, 9.152°E	12.06.2014	0	21.1	Riverine endmembers
E1	53.423°N, 10.339°E	17.07.2014	0.27	22.6	
E2	53.841°N, 8.89167°E	14.06.2014	9.4	18.4	Estuarine samples
E3	53.95483°N, 8.6395°E	14.06.2014	17.4	17.9	
W2	53.534°N, 8.575°E	07.07.2014	18.1	19.5	
M1	54.3355°N, 7.7075°E	15.06.2014	32.9	12.8	Marine endmember

## DOC, TDN and DON analysis

Concentrations of DOC and total dissolved nitrogen (TDN) in filtered water were determined by high temperature catalytic oxidation (HTCO) and subsequent nondispersive infrared spectroscopy and chemiluminescence detection (TOC-VCPN analyzer, Shimadzu). For the determination of solid-phase extractable DOC (DOC_SPE_, pH 2 and pH 8) and DON (DON_SPE_, pH 2), 50 μL (250 μL for DON_SPE_, pH 8) of each methanol extract was evaporated under N_2_ and subsequently redissolved in 6.5 mL ultrapure water. All samples were acidified in the auto sampler (0.1 M HCl suprapur, Merck) and purged with O_2_ for > 5 min to remove inorganic carbon. Performance of the instrument was recorded by the analysis of potassium hydrogen phthalate standard solutions and the deep-sea reference samples (DSR, Hansell research lab). Final DOC and TDN concentrations are average values of triplicate measurements. If the standard variation or the coefficient of variation of DOC values exceeded 0.1 μM or 1%, respectively, up to two additional analyses were performed and outliers were eliminated. For DON, outliers of triplicate measurements were eliminated manually. The accuracy was ± 5% for DOC and ± 7% for DON.

### ICP-MS analysis

For quantification of DOS_SPE_, DOP_SPE_ and trace elements (^51^V, ^52^Cr, ^59^Co, ^60^Ni, ^63^Cu, ^75^As), an inductively coupled plasma mass spectrometer (ICP-MS, Element 2, Thermo Fisher Scientific) was equipped with a desolvation nebulizer (Apex Q, Elemental Scientific), a platinum guard electrode, and nickel sampler and skimmer cones. Prior to ICP-MS analysis, 50 μL of the extract was evaporated with N_2_ gas and redissolved in 2 mL nitric acid (1 M, bidestilled, Merck). 50 μL of ^103^Rh (50 ppb in the spike solution) were added as internal standard. The samples were sonicated for 10–15 min to ensure that all DOM was redissolved. The instrument was tuned daily for optimized plasma conditions and accurate mass calibration with a multi-element tuning solution (~0.1 ppb in MilliQ). Signals of ^32^S and ^75^As were recorded in a resolution of 4000 m/Δm, whereas all other elements were recorded in a resolution of 2000m/Δm, for which the instrument was modified to achieve a flat top peak shape (higher precision). Nitric acid (1 M, double destilled, Merck) was used for analysis blank. If the blank values for SPE were higher than the limit of detection (LOD), the extract concentrations were corrected for the respective blanks. Calibration standards for trace elements were prepared in concentrations of 0.001, 0.01, 0.05, 0.1, 0.5, 1, 5, 10, 50, 100, and 250 μg L^-1^ from a stock solution (100 mg L^-1^, multi-element-standard, nonmetals, Spetec). Limits of detection (according to the German industry standard; DIN 32645) are given in S1 Table.

### Trace element analysis of filtered seawater samples

We analyzed ^59^Co and ^63^Cu in original (filtered) water samples. All labware used for analysis was pre-cleaned according to Dick et al. [40]. Samples for dissolved trace metal analysis were acidified to pH 1.75 using bidistilled HNO_3_. As organic ligands form complexes with Co and Cu which are relatively blind to the chelating resin and therefore pass through it without being extracted, one half of each sample was UV digested to analyze the total amount of Co and Cu [41]. For UV digestion, samples were filled into pre-cleaned PFA bottles and UV-oxidized for 1.5 h using a 450 W photochemical UV power supply from ACE GLASS (photochemical lamp number 7825; Power Supply number 7830). Two procedural blanks were processed the same way. Prior to analysis, each sample was spiked with Indium as internal standard (final concentration 1 ppb). The multi-element analyses of water samples were performed using a seaFAST system (Elemental Scientific Inc.) as described in Hathorne et al. [42] coupled to ICP-MS (Element 2, Thermo Fisher Scientific). The ICP-MS was optimized every day to achieve oxide forming rates below 0.3%. The ICP-MS was modified to achieve a flat top peak shape (higher precision) with a resolution of R = 2000. Quantification limits are 0.35 ng L^-1^ for ^59^Co and 7.35 ng L^-1^ for ^63^Cu. To assess the accuracy and precision of the method, the NASS-6 reference standard was analyzed in a 1:2 dilution at the beginning, in between and at the end of a batch run (n = 5). For Cu, we found 208 ng L^-1^ with a relative standard deviation of 1.5% (certified 248 ± 25 ng L^-1^). The Student *t*-Test was used to compare our values to the certified ones (n = 5, 99% significance level) and showed no significant difference. Within the GeoRem database Takano et al. reported Cu concentrations of 224 ng L^-1^, which is in agreement with our findings [43, 44]. Co is not certified for the NASS-6 standard. For this, only an indicative value is given in the certificate (15 ng L^-1^). We found 11.2 ng L^-1^ with a precision of 0.6%.

### RP-HPLC analysis

Reversed phase high performance liquid chromatography (RP-HPLC) was performed on a LaChrom Elite^TM^ HPLC-system (Hitachi) equipped with a pump (L-2130), autosampler (L-2200), column oven (L-2300), diode array detector (DAD, L-2450, 210 nm) and fluorescence detector (L-2485; excitation: 260 nm, emission: 430 nm) according to Koch et al. [45]. Of each methanol extract, 100 μL extract were diluted with 400 μL ultrapure water. Original water samples were analyzed without any pre-treatment. For each analysis, 30 μL of the methanol extract and 95 μL of original samples were injected respectively. The separation based on polarity (and molecular size) was performed using a reversed-phase column (4μm Hydro-RP 80 Å, 250 x 4.60 mm; Phenomenex, Synergi) and a solvent gradient (0 to 70 min) from 100% ultrapure water (adjusted with low-concentrated NaOH (Merck, suprapur) to pH 7) to 100% methanol (Merck, LiChrosolv, Table 2). Analysis blanks were performed with 100 μL methanol and 400 μL ultrapure water for the analysis of the extracts and ultrapure water only for the analysis of the original samples respectively. Peak areas of the samples were corrected for the respective blanks. Based on RP-HPLC analyses, we differentiated two major DOM_SPE_ fractions: the polar water-soluble fraction with a retention time < 24 min and the non-polar methanol soluble fraction with a retention time > 24 min. We calculated polar/non-polar ratios (DOC_pol_/DOC_non-pol_) to elucidate changes in DOM polarity with changing salinity. Since nitrate and nitrite can absorb in the DAD_210nm_ range, only fluorescence data were used for the evaluation of the DOM polarity characteristics in the original samples.

**Table 2:** Gradient for the chromatographic run. Water (adjusted to pH 7) and methanol were used as eluents.

Time [min]	Water [%]	Methanol [%]	Flow rate [mL min^-1^]
0	100	0	0.2
6	100	0	0.2
20	0	100	0.4
35	0	100	0.4
45	100	0	0.3
55	100	0	0.2
65	100	0	0.2

### Data evaluation and statistical analysis

Outliers in quadruplicate measurements of DOM_SPE_ concentrations were defined by Grubbs Test with a significance level of α = 0.5. Statistical analysis was performed with the software *R*: Analysis of variances between the riverine (E1, W1) and marine (M1) endmember samples) was performed with Mann-Whitney-U-Test (function “wilcoxon.test” in the software *R*). Correlation of changing DOM, DOM_SPE_ and metal-DOM_SPE_ concentrations, respectively, with changing salinity was analyzed using correlation analysis (function “cor.test” in *R*). The variables were not manipulated prior to statistical analysis.

## Results

### Changes in DOM concentration and stoichiometry

**Fig 3:**
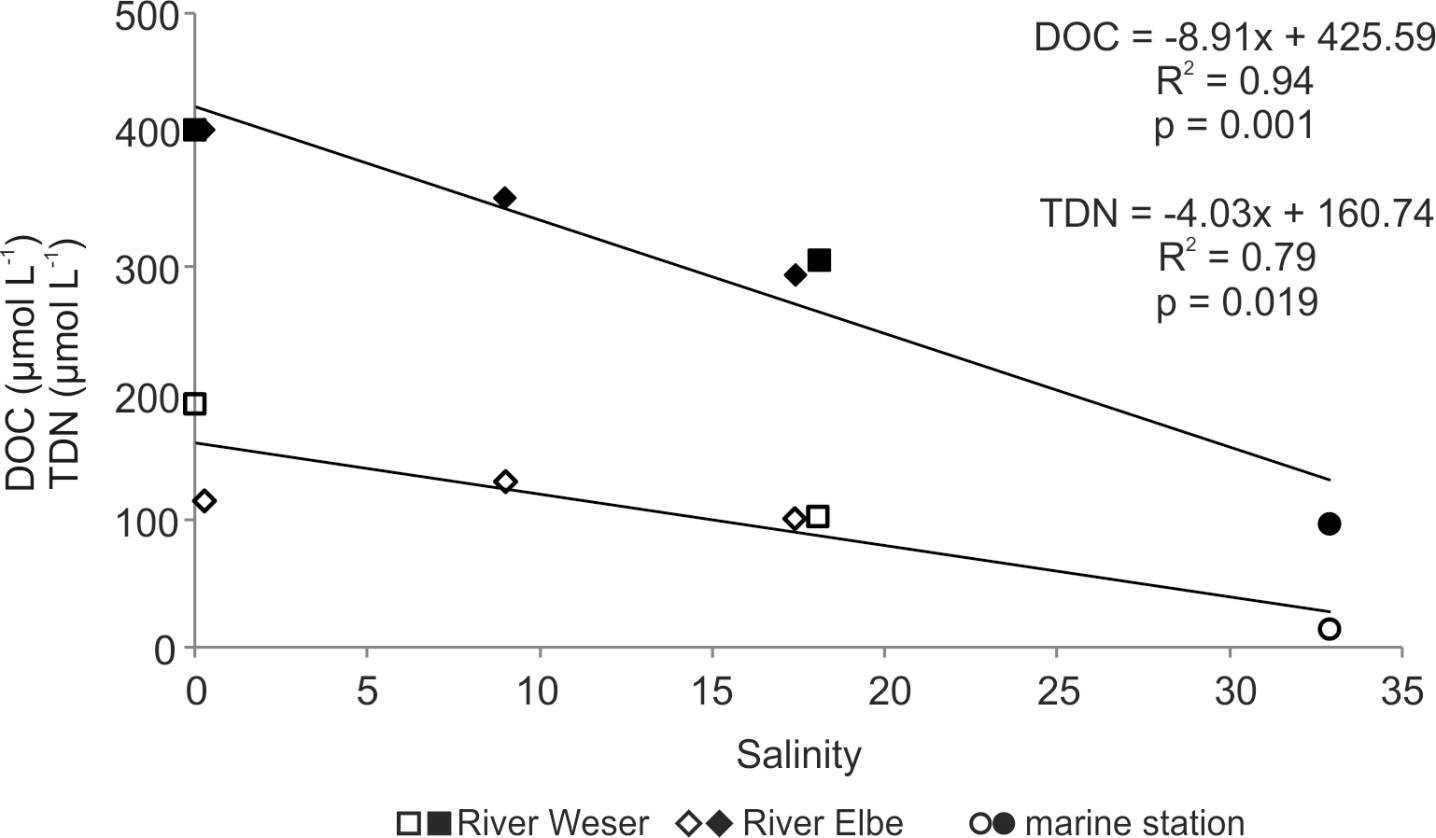
Changes of DOC (filled symbols) and TDN (unfilled symbols) concentrations of original water samples with salinity. Symbols represent sampling locations: River Weser (W1, W2; squares), River Elbe (E1 - E3; diamonds) and the marine station (M1; circles).

The original DOC and TDN concentrations in the water samples decreased with increasing salinity from a maximum of 407 μmol L^-1^ and 190 μmol L^-1^, respectively, in riverine water (W1) to 293 μmol L^-1^ DOC and 101 μmol L^-1^ TDN in estuarine water (E3), and 97 μmol L^-1^ DOC and 14 μmol L^-1^ TDN in the marine sample (M1; Fig 3, Table 3).

**Table 3:** Average DOM_SPE_ concentrations and molar DOC_SPE_/DON_SPE_, DOC_SPE_/DOP_SPE_, DOC_SPE_/DOS_SPE_ ratios at the stations with different salinities. The values are averages of quadruplicate measurements (except of DOC and TDN). Stations are ordered by increasing salinity.

Sample	W1		E1		E2		E3		W2		M1	
Salinity	0		0.3		9		17.4		18.1		32.9	
DOC (μmol L^-1^)	407		407		354		293		304		97	
TDN (μmol L^-1^)	190		114		130		101		102		14	
pH	2	8	2	8	2	8	2	8	2	8	2	8
DOC_SPE_ (μmol L^-1^)	144 ± 4	37 ± 1	143 ± 6	31 ± 1	138 ± 2	33 ± 1	109 ± 4	28 ± 1	116 ± 2	30 ± 1	31 ± 1	10 ± 1
DON_SPE_(μmol L^-1^)	5.9 ± 0.2	1.4 ± 0.1	6.3 ± 0.1	1.3 ± 0.0	9.4 ± 0.1	1.4 ± 0.1	5.7 ± 0.4	1.2 ± 0.1	5.3 ± 0.3	1.4 ± 0.2	1.4 ± 0.0	0.5 ± 0.1
DOC_SPE_/DON_SPE_	25 ± 1	26 ± 2	23 ± 1	24 ± 0	15 ± 0	24 ± 1	19 ± 1	25 ± 2	22 ± 1	23 ± 2	23 ± 1	20 ± 3
DOS_SPE_ (μmol L^-1^)	1.35 ± 0.10	0.35 ± 0.03	1.44 ± 0.02	0.30 ± 0.01	1.39 ± 0.10	0.34 ± 0.01	1.80 ± 0.59-	0.43 ± 0.12	0.89 ± 0.02	0.28 ±0.01	0.19 ± 0.02	0.14 ± 0.03
DOC_SPE_/DOS_SPE_	107 ± 5	110 ± 11	100 ± 3	105 ± 1	100 ± 6	98 ± 2	70 ± 26	71 ± 17	131 ± 4	109 ± 2	162 ± 17	80 ± 15
DOP_SPE_ (nmol/L)	17.1 ± 1.7	5.3 ± 0.5	20.7 ± 0.7	12.4 ± 0.3	14.8 ± 0.9	6.5 ± 0.6	17.3 ±1.8	6.9 ± 0.3	18.0± 0.2	6.4 ± 0.2	10.5 ± 2.1	5.2 ± 1.6
DOC_SPE_/DOP_SPE_ *10^3^	8.5 ± 0.8	7.2 ± 0.4	7.0 ± 0.4	2.4 ± 0.1	9.4 ± 0.6	5.3 ± 0.5	6.4 ± 0.6	4.2 ± 0.1	6.5 ± 0.2	4.9 ± 0.1	3.1 ± 0.5	2.2 ± 0.5

For DOM_SPE_, significantly higher concentrations were found for all elements in samples extracted at pH 2 compared to those extracted at pH 8 (p < 0.001). Therefore, this section focusses on the results of pH 2 extracts. Data on pH 8 extracts can be found in Tables 3 and 4.

**Table 4:** Average metal-DOM_SPE_ concentrations and molar DOC_SPE_/metal-DOM_SPE_ ratios (/10^5^) at the stations. The values are average values of quadruplicate measurements. All concentrations are given in nmol L^-1^.

Sample	W1		E1		E2		E3		W2		M1	
pH	2	8	2	8	2	8	2	8	2	8	2	8
V-DOM_SPE_	0.12 ± 0.04	0.04 ± 0.03	0.11 ± 0.00	≤ LOD	0.27 ± 0.03	-	0.09 ± 0.00	-	0.25 ± 0.01	0.02 ± 0.01	0.06 ± 0.04	≤ LOD
DOC_SPE_/V-DOM_SPE_	11.5 ±0.3	-	13.0 ±0.5	-	5.2 ± 0.1	-	11.8 ±0.4	-	4.8 ± 0.1	1.7 ±0.1	5.4 ±0.2	-
Cr-DOM_SPE_	0.45 ± 0.04	0.01 ± 0.00	0.90 ± 0.11	0.01 ± 0.00	0.43 ± 0.02	0.08 ± 0.06	0.39 ± 0.04	-	0.65 ± 0.04	0.02 ± 0.00	0.09 ± 0.05	0.05 ± 0.00
DOC_SPE_/Cr-DOM_SPE_	3.2 ± 0.4	38.4 ± 5.2	1.6 ± 0.2	31.9 ± 9.6	3.2 ± 0.2	8.8 ± 8.0	2.9 ± 0.4	-	1.8 ± 0.1	16.7 ± 0.8	3.3 ± 1.0	2.3 ± 0.1
Co-DOM_SPE_	0.25 ± 0.01	0.07 ± 0.01	0.38 ± 0.00	0.09 ± 0.00	0.19 ± 0.02	0.06 ± 0.00	0.13 ± 0.00	0.05 ± 0.00	0.15 ± 0.01	0.06 ± 0.01	0.03± 0.00	0.02 ± 0.01
DOC_SPE_/ Co-DOM_SPE_	5.7 ± 0.2	5.7 ± 0.6	3.8 ± 0.2	3.7 ± 0.1	7.4 ± 0.7	5.8 ± 0.4	8.4 ± 0.4	5.7 ± 0.5	7.9 ± 0.7	5.5 ± 0.4	10.3 ± 1.4	6.2 ± 1.5
Ni-DOM_SPE_	0.53 ± 0.05	0.23 ± 0.06	0.59 ± 0.02	0.39 ± 0.01	0.5 ± 0.03	0.44 ± 0.03	0.33 ± 0.04	0.4 ± 0.01	0.31 ± 0.01	0.32 ± 0.01	0.09 ± 0.01	0.09 ± 0.01
DOC_SPE_/ Ni-DOM_SPE_	2.7 ± 0.3	1.8 ± 0.5	2.4 ± 0.2	0.8 ± 0.03	2.8 ± 0.2	0.8 ± 0.1	3.4 ± 0.4	0.7 ± 0.04	3.8 ± 0.2	1.0 ± 0.03	3.7 ± 0.6	1.1 ± 0.3
Cu-DOM_SPE_	4.60 ± 0.39	2.30 ± 0.12	4.13 ± 0.10	1.76 ± 0.11	5.35 ± 0.39	4.46 ± 0.53	3.41 ± 0.41	4.30 ± 0.32	3.58 ± 0.18	3.89 ± 0.41	0.77 ± 0.18	1.40 ± 0.77
DOC_SPE_/Cu-DOM_SPE_	0.3 ± 0.02	0.2 ± 0.01	0.4 ± 0.02	0.2 ± 0.01	0.3 ± 0.02	0.08 ± 0.01	0.3 ± 0.03	0.07 ± 0.01	0.3 ± 0.01	0.08 ± 0.01	0.4 ± 0.09	0.1 ± 0.05
As-DOM_SPE_	0.63 ± 0.06	≤ LOD	0.82 ± 0.06	-	0.83 ± 0.04	-	0.87 ± 0.06	-	0.85 ± 0.08	0.15 ± 0.02	0.77 ± 0.06	0.11 ± 0.01
DOC_SPE_/As-DOM_SPE_	1.2 ± 0.1	-	1.1 ± 0.03	-	1.0 ± 0.02	-	0.8 ± 0.04	-	0.8 ± 0.05	0.5 ± 0.005	0.2 ± 0.01	0.2 ± 0.02

LOD: limit of detection

DOC_SPE_ concentrations of pH 2 extracted samples decreased significantly from 144 ± 4 μmol L^-1^ in the riverine endmember (W1) to 31 ± 1 μmol L^-1^ in the marine endmember sample (M1) (p < 0.01, Table 3). Using the DOC concentrations in the original samples and the DOC_SPE_ concentration, we calculated the DOC extraction efficiencies. The average DOC extraction efficiencies at pH 2 were 36 ± 2%. No significant correlation of the extraction efficiency with salinity was found (p > 0.05).

DON_SPE_ concentrations of pH 2 extracted samples decreased significantly with increasing salinity from 6.3 ± 0.1 μmol L^-1^ in riverine water (E1) to 1.4 ± 0.0 μmol L^-1^ in seawater (M1) (p = 0.01). Differences of the average molar DOC_SPE_/DON_SPE_ ratios of 24 ± 1 and 23 ± 1 were insignificant between pH 2 extracted riverine (E1, W1) and marine (M1) endmember (for pH 8 extracted samples however, they decreased significantly from 25 ± 1 to 22 ± 1 in riverine (E1, W1) and marine (M1) samples (p = 0.01)).

Similar to DOC_SPE_ and DON_SPE_, concentrations of DOS_SPE_ and DOP_SPE_ were higher in pH 2 than in pH 8 extracted samples (p < 0.001, Table 3). DOS_SPE_ concentrations of pH 2 samples decreased significantly from 1.44 ± 0.02 μmol L^-1^ in riverine (E1) to 0.19 ± 0.02 μmol L^-1^ in marine water (p = 0.01). To address the influence of mixing of low and high salinity waters, we normalized DOS_SPE_ and DOP_SPE_ concentrations to DOC_SPE_ concentrations and thus calculated molar DOC_SPE_/DOS_SPE_ ratios and DOC_SPE_/DOP_SPE_ ratios, respectively. Average DOC_SPE_/DOS_SPE_ ratios of pH 2 extracted riverine waters (E1, W1) were 103 ± 5 and increased to 162 ± 17 in the marine sample (p = 0.01). No differences in molar DOC_SPE_/DOS_SPE_ ratios were found between pH 2 and pH 8 extracted samples.

Compared to DOS_SPE_, DOP_SPE_ concentrations of pH 2 samples were two orders of magnitude lower and decreased significantly from 20.7 ± 0.7 nmol L^-1^ to 10.5 ± 2.1 nmol L^-1^ in riverine (E1) and marine water, respectively (p < 0.001). Molar DOC_SPE_/DOP_SPE_ ratios of pH 2 extracted samples decreased significantly from riverine to marine water (p < 0.01).

Thus, average molar C:N:P:S ratios of pH 2 extracted samples were C_106_:N_4_:P_0.013_S_1_ for the riverine endmember (W1) and C_164_:N_7_:P_0.053_S_1_ for the marine endmember.

### Changes in DOM polarity

Using reversed-phase chromatography, we found a good relationship of both fluorescence (260/430 nm) and absorption (210 nm) data (total peak areas) with measured DOC and DOC_SPE_ concentrations (R^2^ = 0.3 and p < 0.01 for DOC concentrations of 0–40 μmol L^-1^ versus UV peak areas and R^2^ = 0.6 and p < 0.001 for DOC concentrations > 100 μmol L^-1^ versus UV peak areas, S1 Fig), confirming that UV absorption in the extracts serves as a suitable predictor of DOC concentration [46]. Lechtenfeld et al. showed that this correlation can also be found in individual chromatographic fractions [46]. In this section, we will use integrated peak areas of fluorescence / adsorption as a proxy for DOC_SPE_ concentration.

Fluorescence data for the original samples confirmed decreasing DOC concentrations (peak areas) with increasing salinity (not shown). For the solid-phase extracted DOM, we found a significant linear correlation of decreasing polar peak areas with increasing salinity (R^2^ = 0.9; p < 0.01), whereas no significant trend was found for non-polar peak areas. Hence, DOC_pol_/DOC_non-pol_ ratios significantly decreased with increasing salinity (R^2^ = 0.7, p = 0.035).

**Fig 4:**
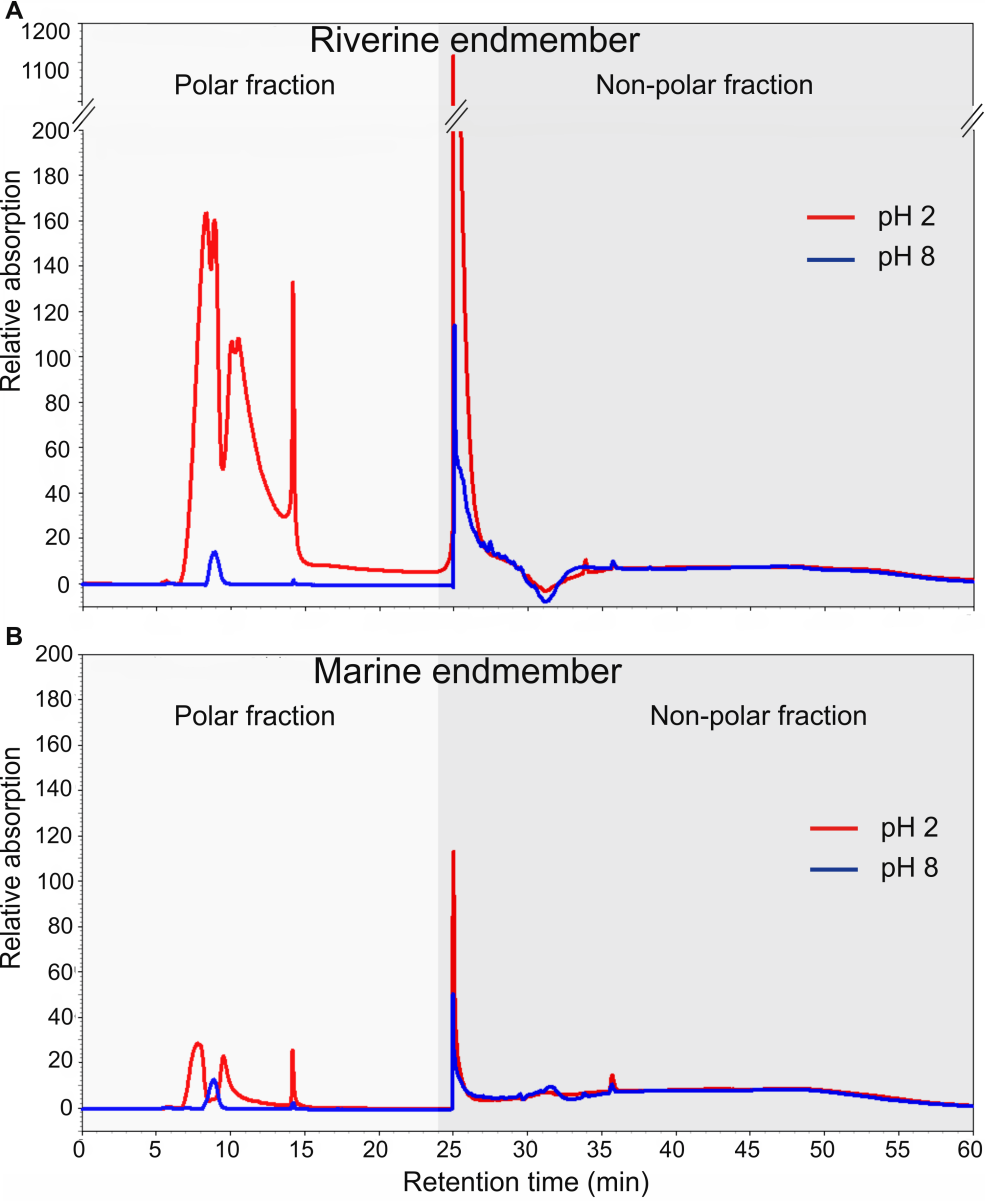
UV absorption chromatograms of riverine and marine methanol extracts at 210 nm. (A) Chromatograms of riverine pH 2 (red) and pH 8 (blue) extracted samples. (B) Chromatograms of marine pH 2 (red) and pH 8 (blue) extracted samples.

In addition, absorption (Fig 4) and fluorescence data (not shown) confirmed significantly higher DOC concentrations in pH 2 compared to pH 8 extracted samples (p < 0.001).

**Fig 5:**
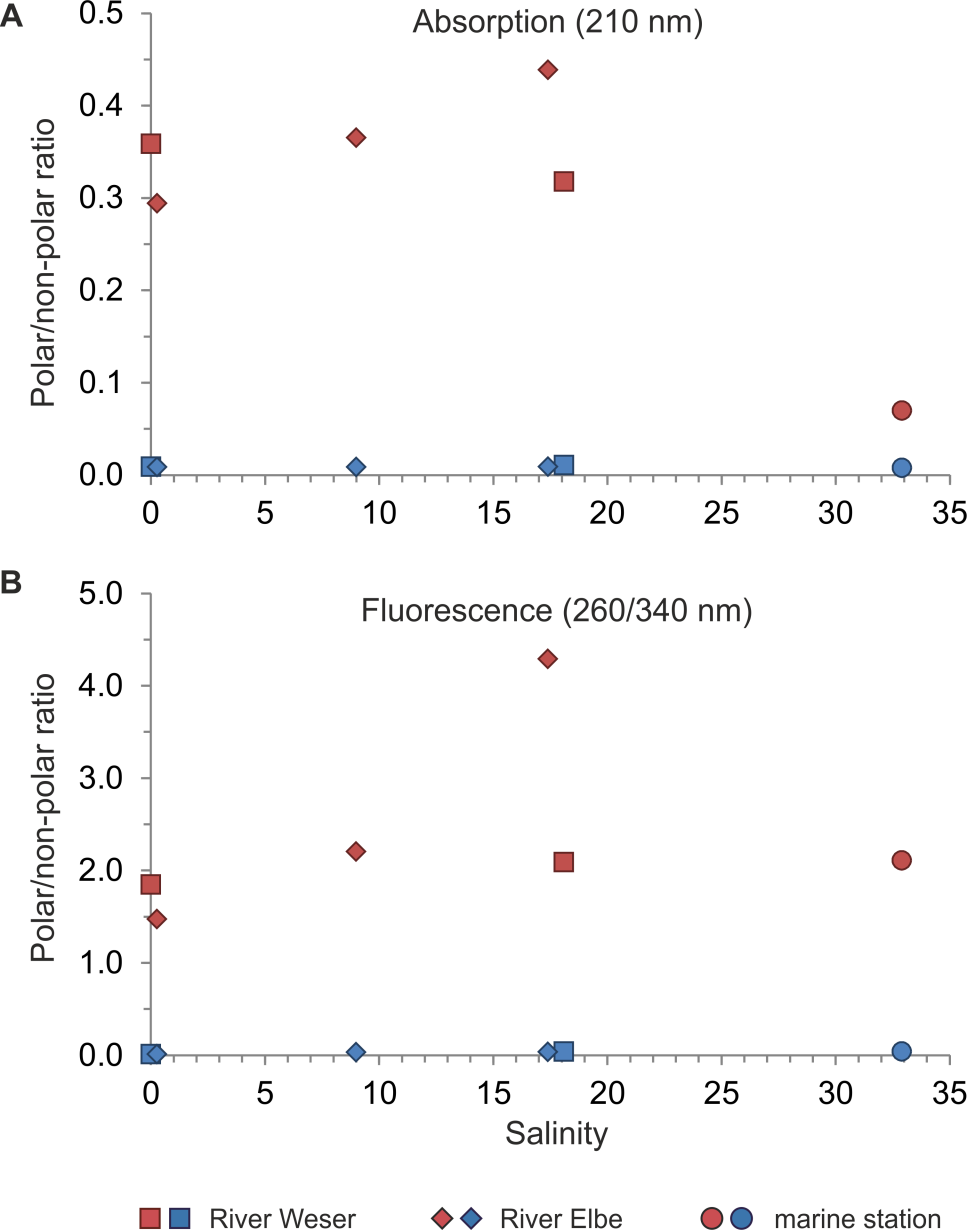
Changes in polar/non-polar peak area ratios of DOM_SPE_ samples with salinity changes. (A) Average polar/non-polar peak area ratios analyzed by UV spectroscopy (DAD_210nm_) versus salinity and (B) average polar/non-polar peak area ratios analyzed by fluorescence spectroscopy (260/340 nm) versus salinity. The pH 2 extracted samples are indicated by red symbols, pH 8 extracted samples by blue symbols. Symbols represent sampling locations: River Weser (W1, W2; squares), River Elbe (E1 - E3; diamonds) and the marine station (M1; circles).

Absorption data showed significantly higher DOC_SPE_ concentrations (peak areas) at low salinity compared to high salinity samples (p < 0.01, Fig 4). However, the DOC_SPE_ concentration (peak areas) in the estuarine samples E3 and W3 (salinity of 17.4 and 18.1, respectively) were similar to sample E2 at salinity 9.4 and thus deviated from the linear regression line (R^2^ = 0.96, p < 0.001). For all extracts, the ratio of DOC derived from polar compounds compared to the total DOC concentration (DOC_pol_/DOC_tot_) was significantly higher in pH 2 compared to pH 8 samples (p < 0.001). In pH 2 extracted samples, DOC_pol_/DOC_tot_ of 0.25 ± 0.02 in riverine samples (W1, E1) was significantly higher compared to 0.06 ± 0.02% in marine samples (p < 0.01). Consequently, we observed a significant decrease of DOC_pol_/DOC_non-pol_ in pH 2 samples from 0.36 ± 0.03 in riverine water (W1) to 0.07 ± 0.02 in marine water (p = 0.01, Fig 5A), whereas no significant changes in pH 8 samples occurred. However, if we focus on Elbe samples only, we observed a relative increase of DOC_pol_/DOC_non-pol_ ratios in estuarine water (Fig 5A).

Fluorescence data showed that DOC_SPE_ concentrations (peak areas) of both pH 2 and pH 8 extracted samples decreased with increasing salinity (p < 0.001). Overall, DOC_pol_/DOC_tot_ of 0.68 ± 0.07 in pH 2 samples was significantly higher compared to 0.03 ± 0.01 in pH 8 samples (p < 0.001). DOC_pol_/DOC_tot_ in both pH 2 and pH 8 extracted samples decreased significantly with increasing salinity (p < 0.001). This results in a significant increase in DOC_pol_/DOC_non-pol_ of 1.7 ± 0.2 to 2.1 ± 0.3 in pH 2 extracted riverine and marine samples, respectively, and from 0.01 ± 0.002 to 0.04 ± 0.01 in pH 8 extracted riverine and marine samples, respectively (Fig 5B). Comparing the concentration of non-polar DOC (DOC_non-pol_) between pH 2 and pH 8 samples, it is noteworthy that in pH 8 samples, DOC_non-pol_ was only about half of the value in pH 2 samples. By contrast, the contribution of the DOC_non-pol_ pool to the absorbance of pH 2 or pH 8 extracted samples was similar.

### Trace metals

Concentrations of solid-phase extractable trace metals (metal-DOM_SPE_) were generally higher in pH 2 compared to pH 8 extracts, with the exception of Ni-DOM_SPE_. In pH 2 extracted samples, Co-DOM_SPE_, Ni-DOM_SPE_, Cu-DOM_SPE_, and Cr-DOM_SPE_ concentrations decreased with increasing salinity, similar to DOC_SPE_ and DOS_SPE_ (Table 4). Although riverine Cu-DOM_SPE_ was significantly higher than in the marine sample (p < 0.05), we found a concentration maximum at E2 followed by a decrease with increasing salinity. V-DOM_SPE_ concentrations also increased in estuarine waters, followed by a decrease in the high salinity marine water, but were highly distributed over all samples. Also, Cr-DOM_SPE_ concentrations were highly distributed. Nevertheless, we could also observe a significant decrease with increasing salinity over all Cr-DOM_SPE_ samples (p < 0.01). No significant differences between riverine and marine endmember concentrations were found for As-DOM_SPE_ and V-DOM_SPE_. Process blanks were below the detection limit for Co-DOM_SPE_, Ni-DOM_SPE_, and Cu-DOM_SPE_. Blanks for As-DOM_SPE_, Cr-DOM_SPE_, and V-DOM_SPE_ were measurable and about factor 2.0 – 5.8 lower for riverine and estuarine samples and by factor 1.2–2.4 lower for marine samples compared to the corresponding metal-DOM_SPE_ concentrations in the samples. To facilitate the subsequent comparison of the relative metal and sulfur content in DOM, we used C/metal ratios (similar to C/S ratios).

**Fig 6:**
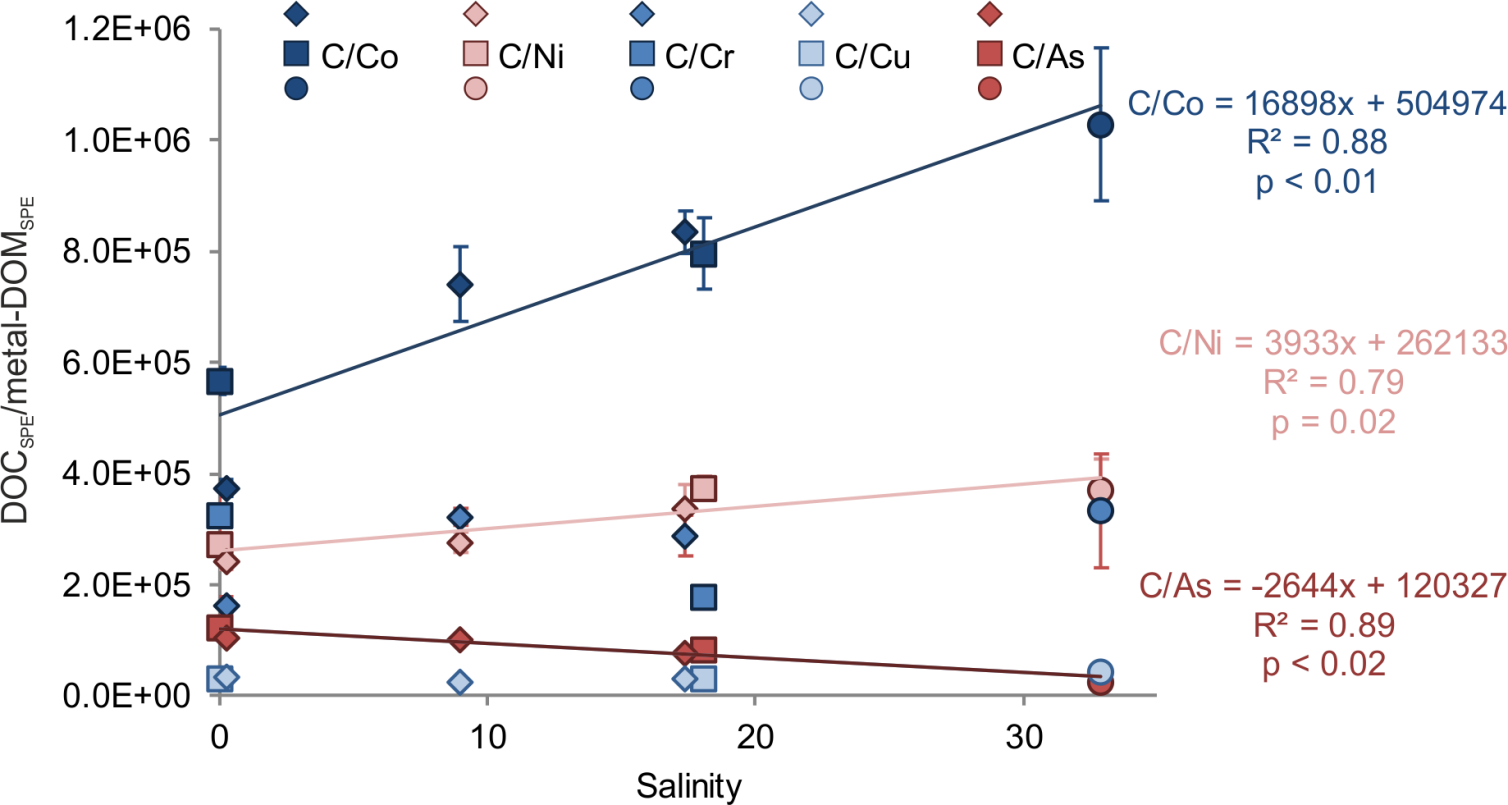
Changes in trace-metal stoichiometry with salinity. Linear correlation of average molar DOC_SPE_/metal-DOM_SPE_ ratios of pH 2 extracted samples (except of DOC_SPE_/Cr-DOM_SPE_, DOC_SPE_/Cu-DOM_SPE_, and DOC_SPE_/V-DOM_SPE_) versus salinity. Symbols represent sampling locations: River Weser (W1, W2; squares), River Elbe (E1—E3; diamonds) and the marine station (M1; circles).

Similar to DON_SPE_, DOP_SPE_ and DOS_SPE_, the concentrations of metal-DOM_SPE_ were normalized to DOC_SPE_ (Table 4). The decrease in Co-DOM_SPE_ and Ni-DOM_SPE_ concentrations with increasing salinity was a more sensitive function of salinity than that of DOC_SPE_ decrease, resulting in a significant linear increase of the molar DOC_SPE_/Co-DOM_SPE_ (p < 0.01) and DOC_SPE_/Ni-DOM_SPE_ ratios (p = 0.01 Fig 6). In contrast, DOC_SPE_/As-DOM_SPE_ ratios decreased significantly with increasing salinity (p < 0.01). No significant differences between riverine and marine samples were found for DOC_SPE_/Cr-DOM_SPE_, DOC_SPE/_Cu-DOM_SPE_ and V-DOM_SPE_.

In pH 8 extracted samples, most concentrations of As-DOM_SPE_ and V-DOM_SPE_ were below the detection limit (LOD; cf. Table 4). Additionally, a high variance in quadruplicate measurements was found for the low concentrations of all trace metals in the marine samples, therefore those results were excluded from further discussion. Data of metal-DOM_SPE_ concentrations and DOC_SPE_/metal-DOM_SPE_ ratios of pH 8 extracts can be found in Table 4.

**Fig 7:**
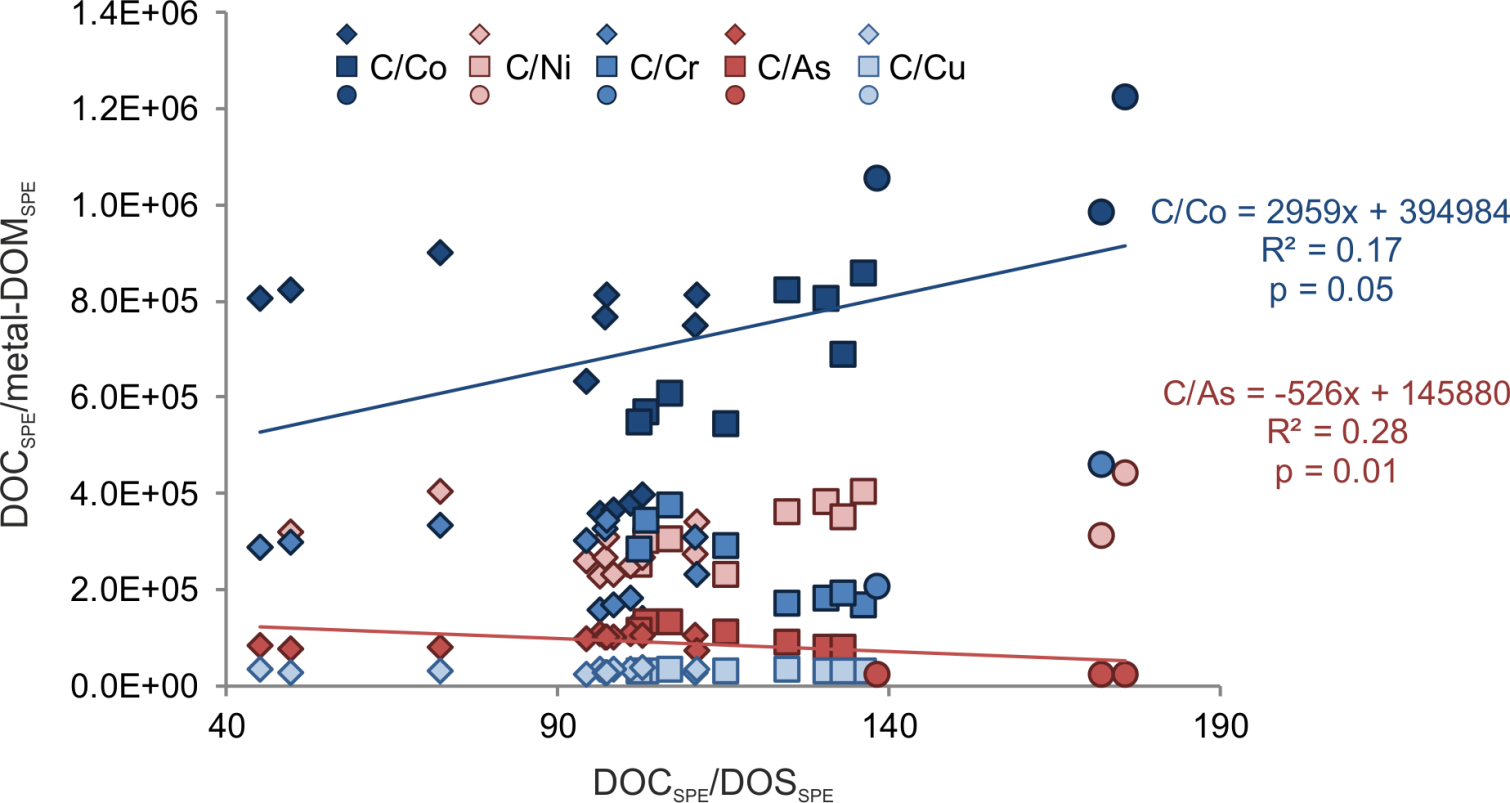
Correlation of trace metal and DOS stoichiometry. Correlation of DOC_**SPE**_/metal-DOM_**SPE**_ versus average DOC_**SPE**_/DOS_**SPE**_ of pH 2 extracted samples. Linear correlation of DOC_**SPE**_/Co-DO_**SPE**_ and DOC_**SPE**_/As-DOM_**SPE**_ is shown. Symbols represent sampling locations: River Weser (W1, W2; squares), River Elbe (E1 - E3; diamonds) and the marine station (M1; circles).

To verify if DOS_SPE_ correlates with trace metals, we plotted DOC_SPE_/metal-DOM_SPE_ ratios versus DOC_SPE_/DOS_SPE_ ratios of pH 2 extracted samples (Fig 7). We observed a significant negative linear correlation of DOC_SPE_/As-DOM_SPE_ (R^2^ = 0.28, p = 0.01) with DOC_SPE_/DOS_SPE_ and a positive linear correlation for DOC_SPE_/Co-DOM_SPE_ (R^2^ = 0.17, p = 0.05). DOC_SPE_/Cr-DOM_SPE_, DOC_SPE_/V-DOM_SPE_, DOC_SPE_/Ni-DOM_SPE_, and DOC_SPE_/Cu-DOM_SPE_ showed no correlation with DOC_SPE_/DOS_SPE_ (Fig 7). For pH 8 extracted samples, no significant correlation was found.

**Fig 8:**
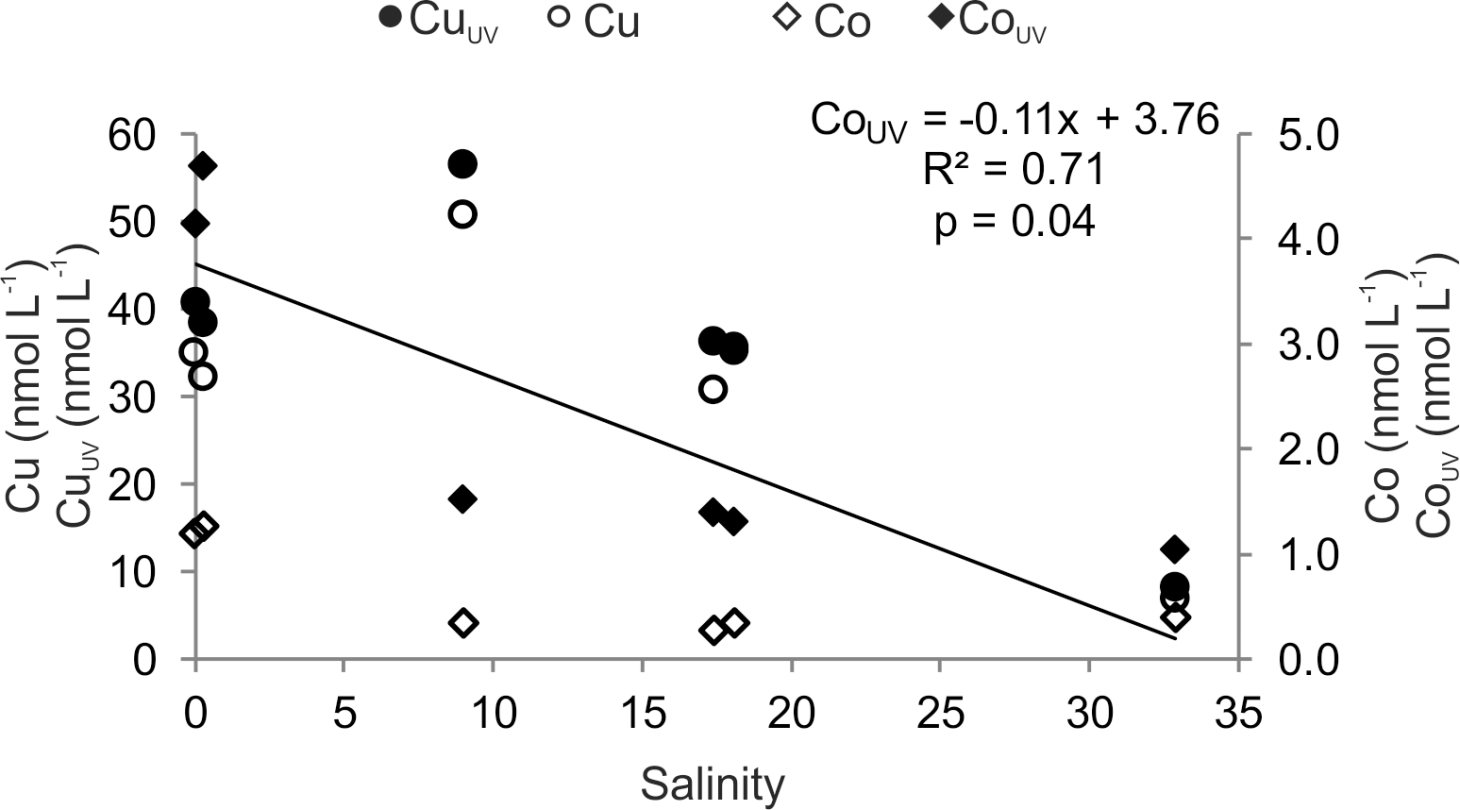
Cu (circles) and Co (diamonds) concentrations with changing salinity. Symbols and correlation line represent pre-treatment conditions of original samples: with UV digestion (filled symbols, solid line) and without UV digestion (unfilled symbols).

To assess the extraction efficiencies for metal-DOM, we determined the trace metal concentrations of Co and Cu in original seawater and compared them with Cu-DOM_SPE_ and Co-DOM_SPE_ (Table 5). Co and Cu concentrations in original water samples also decreased with increasing salinity (Fig 8, Table 5). A significant linear correlation with salinity was found for UV-treated Co (Co_UV_) concentrations only (Fig 8). The application of UV digestion led to 300–500% higher trace element concentrations compared to the untreated samples. Co extraction efficiencies were generally lower for pH 8 extracted samples compared to pH 2 extracted samples. Average extraction efficiencies of pH 2 extracted Co-DOM_SPE_ were 8–45% without UV digestion of original samples and 3–12% with UV digestion, respectively. Co extraction efficiencies of riverine samples were significantly higher compared to marine samples (p < 0.001). However, no correlations with salinity were observed. For pH 8 extracted samples, average Co-DOM_SPE_ extraction efficiencies were 5–19% and 2–4% for samples without and with UV digestion, respectively. No differences between riverine and marine samples were found. Average extraction efficiencies of pH 2 extracted Cu-DOM_SPE_ were 10–13% without UV digestion of original samples and 9–11% with UV digestion. For pH 8 extracted samples, Cu-DOM_SPE_ extraction efficiencies were 5–20% and 6–17% for samples without and with UV digestion, respectively. In contrast to Co-DOM_SPE_, the extraction efficiencies of pH 8 extracted Cu correlated linearly with salinity (R^2^ = 0.98, p < 0.001). Interestingly, Cu extraction efficiencies of pH 2 extracted samples showed no differences with increasing salinity, whereas Cu extraction efficiencies of pH 8 extracted samples increased with increasing salinity (Table 5).

**Table 5:** Dissolved Co and Cu concentrations in original water samples and corresponding average solid phase extraction efficiencies (extr. eff.).

		without UV digestion				with UV digestion			
Sample	Salinity (psu)	Co conc. (nmol L^-1^)	Co extr. eff. pH 2/pH 8 (%)	Cu conc. (nmol L^-1^)	Cu extr. eff. pH 2/pH 8 (%)	Co_UV_ conc. (nmol L^-1^)	Co_UV_ extr. eff. pH 2/pH 8 (%)	Cu_UV_ conc. (nmol L^-1^)	Cu_UV_ extr. eff. pH 2/pH 8 (%)
W1	0	1.20	21/6	35	13/7	4.143	6/11	41	11/6
E1	0.3	1.26	30/7	32	13/5	4.701	8/11	39	11/5
E2	9	0.35	54/17	51	11/9	1.526	12/9	57	9/8
E3	17.4	0.27	49/19	31	11/14	1.399	9/9	36	9/12
W2	18.1	0.35	42/16	36	10/11	1.308	11/10	35	10/11
M1	32.9	0.40	8/5	7	11/20	1.037	3/9	8	9/17

## Discussion

### Stoichiometry and polarity characteristics of dissolved organic matter

The high DOM concentrations in the riverine endmember samples of Weser and Elbe River emphasized the importance of rivers as a DOM source to the coastal oceans as previously shown for many other regions from riverine to marine waters [47–50]. DOC concentrations in the estuarine and marine samples (E2, E3, M1; Table 3) were typical for the German Bight (76–209 μmol L^-1^ [51]). DOC_SPE_, DON_SPE_, and DOS_SPE_ concentrations as well as DOC_SPE_/DON_SPE_ and DOC_SPE_/DOS_SPE_ ratios are in accordance with values published for the North Sea [52] and Atlantic surface waters [3, 53]. Unlike the typical decrease of DOC/DON ratios from land to sea in estuaries (e.g. [14]), our pH 2 extracted samples showed almost constant molar DOC_SPE_/DON_SPE_ ratios, probably due to an inefficient extraction of nitrogen compared to carbon [54, 55]. Ratios of pH 8 extracted samples were similar, but reflected the typical decrease of molar DOC_SPE_/DON_SPE_ ratios with increasing salinity. Molar DOC_SPE_/DOS_SPE_ ratios were higher and molar DOS_SPE_/DON_SPE_ ratios lower in the riverine compared to the marine endmembers. Our average DOC_SPE_/DOS_SPE_ ratios were 100 ± 3 and 107 ± 5 in the riverine endmember samples. Previous studies on DOS in freshwater and other aquatic systems are very scarce. Houle et al. found DOS concentrations of ~ 5.8 μmol L^-1^ (~ 185 μg L^-1^) and DOC/DOS ratios of ~ 122 in several southwestern Québec lakes [56], values that are higher than those in our riverine samples. However, these lakes are influenced by terrestrial DOM with high DOC and DOS concentrations from forest soils. Nevertheless, spatial differences can also cause differences in DOS concentrations. Some information on sulfur exists also for soil and particulate organic matter (POM), e.g. [57, 58]. Based on a molar C/S ratio of 119 for POC [57], the global flux of DOS and POS from rivers to the ocean accounts for 8 Tg S a^-1^ [3], a value that would be ~15% higher if we use the C/S ratio from this study. Our numbers for the molar DOC_SPE_/DOP_SPE_ ratios were very high compared to other studies [59], presumably due to inefficient DOP extraction for the cartridges used. However, this is speculative due to the limited available data.

The chromatographic data reflected also lower DOC_SPE_ concentrations at higher salinity. Since fluorescence represents only a small fraction of DOM_SPE_, we focus on the absorbance data. In contrast to samples from the Weser River, DOC_pol_/DOC_non-pol_ ratios of riverine and estuarine samples of the Elbe River were significantly higher at higher salinity. Possible reasons might be differences in DOM sources or different residence times. Over all samples, however, no significant changes in DOC_pol_/DOC_non-pol_ ratios occurred within the group of pH 2 extracted riverine and estuarine samples (W1, W2, E1—E3). Only for the marine endmember, a significant lower DOC_pol_/DOC_non-pol_ ratio was found. Riverine DOM is dominated by terrestrial sources, characterized by high polarity due to a high number of carboxyl groups, elevated C/N ratios and higher contribution of aromatic components (e.g. lignin and its degradation products) [60, 61] (resulting in higher DOC_pol_/DOC_non-pol_ ratios compared to marine samples, as found also in this study). DOM in marine samples, in contrast, originates mainly from phytoplankton and its degradation products and only 0.7–2.4% appears to be from terrestrial sources [62]. In estuaries, mixing of both, riverine and marine DOM occurs and results in changes of the DOM pool composition: with increasing salinity, molecular weight, carbohydrate and heteroelement content of DOM increases [6, 63], while DOM aromaticity decreases [64]. The highly significant correlation of decreasing DOM_SPE_ concentration with increasing salinity reflects mixing of riverine and marine DOM. However, due to the limited number of samples, precise statements about deviations from conservative mixing, as previously reported (e.g. [50]), would be speculative. To assess if all DOM_SPE_ compounds decreased in a similar range with increasing salinity, we compared the relative changes of DOC_SPE_, DOS_SPE_ and DON_SPE_ endmember concentrations in riverine and marine samples_._ We found a similar reduction in DOC_SPE_ and DON_SPE_ concentrations of ~77 ± 1% and a reduction in DOS_SPE_ concentration of 86 ± 1%. These changes indicate that DOS_SPE_ decreased faster than DOC_SPE_ and DON_SPE_. This is in accordance with previous studies in the oceanic water column, showing preferential depletion of sulfur (and phosphorous) relative to carbon [3, 59]. Similar to sulfur, nitrogen is also removed preferentially to carbon [3]. It might be possible, that in our study, the fast decrease in DON_SPE_ is masked by other DON_SPE_ sources along the estuary (such as agricultural and industrial sources, benthic flux, or microbial activity).

Although the mixing of DOM-rich riverine freshwater with marine water in estuaries is the major factor controlling DOM distribution and composition [48, 50, 65, 66], relative changes in DOM stoichiometry indicate processes beyond estuarine mixing. Different sources and sinks control the amount, composition and reactivity of DOM in aquatic environments: biological release, phyto and zooplankton mediated processes [67], decomposition of riverine DOM by marine bacteria [68], photo-bleaching and photo-degradation [69–72] as well as flocculation processes and sorption to sediments [47, 48, 73]. All of these processes might occur simultaneously, and it remains a major challenge to quantify the influence of each process on DOM composition.

### Trace metal complexation and DOM composition

Co-DOM_SPE_, Ni-DOM_SPE_, and Cu-DOM_SPE_ concentrations decreased with salinity, a result of mixing of trace metal-rich riverine water with trace metal-poor marine water. Higher trace-metal concentrations in the riverine extracts suggest a terrestrial/benthic source for dissolved trace metals [74, 75] and/or differences in trace-metal/organic matter composition. Although statements about conservative or non-conservative mixing would be speculative due to the limited number of samples, we assume that other factors additionally to mixing must occur. Normalization of trace metals to carbon allows us to analyze differences between the decreases in DOC_SPE_ and metal-DOM_SPE_ concentrations. Unlike As-DOM_SPE_ and Cu-DOM_SPE_, Co-DOM_SPE_ and Ni-DOM_SPE_ decreased disproportionately compared to DOC_SPE_, similar to DOS_SPE_. The rapid decrease of trace metal concentrations in the estuarine mixing zone is consistent with previous studies [76, 77] and reasons might be (i) changes in trace metal and/or DOM sources, (ii) changes in DOM quality (polarity) and (iii) consumption. Some trace metals (e.g. V, Cr, and Cu) however, increased in estuarine waters and decreased at high salinity in the marine water.

The distributions of Co-DOM_SPE_, Ni-DOM_SPE_, Cu-DOM_SPE_, and Cr-DOM_SPE_ concentrations with increasing salinity followed that of DOC_SPE_ and DOS_SPE_ concentrations, which implies that they are complexed with organic matter (e.g. via carboxylic, hydroxamate, or thiol groups), whereas V-DOM_SPE_, and As-DOM_SPE_ distributions lead to assume a lower affinity for organic matter. From a study in the Northeast Pacific Ocean, it was estimated that > 99% of total dissolved Cu in surface water is associated with strong organic complexes [78]. Complexation of Co und Cu is further indicated by relatively high extraction efficiencies for both trace metals. We assume that trace metals, which are not organically complexed would most likely not be captured by our extraction method. The PPL sorbent has previously been shown to achieve high recovery rates for organic Cu [79].

The importance of organometallic complexes in DOM is supported by UV digestion prior to seaFAST analysis yielding 100–120% higher Cu concentrations and up to 300–500% higher Co concentrations in the original sample. In previous studies, Co concentrations increased also but only by 50–160% [41, 80]. These differences could be explained by spatial differences in the availability and composition of organic ligands. Since UV treatment/oxidation is used to destroy even very strong metal-organic complexes, the increase in Co and Cu concentrations after UV digestion indicates that a major part of Cu and Co in aquatic samples is organically complexed.

To explore the role of organic sulfur in organometallic complexes, we compared the values of metal-DOM_SPE_/DOC_SPE_ and metal-DOM_SPE_/DOS_SPE_ ratios in the pH 2 extracted riverine (W1) and marine (M1) endmember samples and found an increase in the following order: Cu > As > Ni > Cr > Co and As ≥ Cu > Ni > Cr > Co, respectively. This order is consistent with the Irving-Williams order, which has been used to compare the affinity of (colloidal) trace-metals to organic ligands [76, 81]. It is true for both, the affinity of trace metals to DOC_SPE_ and to DOS_SPE_. According to our results, Cu has a higher affinity to (S-containing) organic ligands than Co and Cr irrespective of the salinity. Comparing the metal-DOM_SPE_ concentrations in riverine and marine endmember samples (Table 4), we can calculate the relative changes of metal-DOM_SPE_ concentrations as it has been done similarly for DOC_SPE_ and DOS_SPE_. We found a relative decrease of metal-DOM_SPE_ with increasing salinity in the order Co (90%) > Cr (87%) > Ni (84%) > Cu (82%). Those differences in relative changes of the trace metal concentrations with increasing salinity are similar to DOS_SPE_ and DOC_SPE_ concentrations and cannot be explained by mixing alone. In fact, different transformation and removal processes (as mentioned in the introduction) can influence DOM concentration. The order in relative changes of metal-DOM_SPE_ concentrations with increasing salinity reflects again the Irving-Williams order, indicating that a higher relative decrease in metal-DOM_SPE_ concentration consequently reflects lower affinity to organic ligands. The stronger the affinity of trace metals to organic ligands, the more resistant are the metal-organic complexes against degradation processes.

Trace metal complexation to organic sulfur groups is further supported by the positive correlation of the ratio of DOC_SPE_/Co-DOM_SPE_ with DOC_SPE_/DOS_SPE_ (Fig 7). Thus, we found indication for a correlation of Co and sulfur. Comparatively little is known about organic complexation of cobalt in aquatic environments. Studies in the Mediterranean Sea and the Scheldt Estuary suggest partial, but strong complexation of Co to organic ligands [82–84]. In organisms, Co and sulfur are coupled via the biosynthesis pathway of methionine: the enzyme methionine synthase is responsible for the regeneration and remethylation of methionine from homocysteinie. In some microorganisms (e.g in *E*. *coli*), this enzyme requires the Co-containing cobalamin (vitamin B12) as a cofactor [85].

Following the Irving-Williams order, we assume the affinity of Ni to organic carbon and sulfur groups to be between that of Cu and Co. It is known that about 10 – 60% of Ni in coastal and marine waters is bound by organic ligands [20, 86, 87]. However, it is unclear whether S-containing organic ligands play a role in nickel complexation and we did not find a significant correlation of DOC_SPE_/Ni-DOM_SPE_ with DOC_SPE_/DOS_SPE_ ratios in our samples.

Although our results suggest a higher affinity of Cu to sulfur than of Co (Irving-Williams order) we could not find a linear correlation of Cu-DOM_SPE_ with DOS_SPE_. It is known that dissolved Cu in different aquatic environments is organically complexed by thiols (e.g. [19]). Laglera and van den Berg analyzed copper-thiol complexes in estuarine waters of the Scheldt River, the Netherlands, and found a decrease in copper-thiol complex stability with increasing salinity [19]. However, thiol concentrations in marine waters are usually very low (< 10 nmol L^-1^) [88–90]. Comparing these concentrations with the calculated minimum DOS concentration of 0.34 μmol L^-1^ in original seawater of the upper East Atlantic Ocean [3], it turns out that thiols contribute to only < 3% of the DOS pool.

We can summarize the differences in riverine and marine trace metal containing DOM_SPE_ by their average molar ratios to be (C_107_N_4_P_0.013_S_1_)_1000_V_0.05_Cr_0.33_Co_0.19_Ni_0.39_Cu_3.41_As_0.47_ in the riverine endmember (W1) and (C_163_N_7_P_0.055_S_1_)_1000_V_0.05_Cr_0.47_Co_0.16_Ni_0.07_Cu_4.05_As_0.58_ in the marine endmember. Compared to the extended Redfield ratio by Ho et al. of (C_95_N_12_P_0.8_S_1_)_1000_Cu_0.29_Co_0.15_ for marine phytoplankton [91], we found a considerably higher DOC_SPE_/DOS_SPE_ ratio in the marine endmember sample, presumably as a result of a more advanced state of degradation. Additionally, we found lower DOC_SPE_/Cu-DOM_SPE_ and DOC_SPE_/Co-DOM_SPE_ ratios in our samples compared to marine phytoplankton.

### Influence of salinity and sample pre-treatment on extraction and trace element complexes

The DOC extraction efficiencies of 36 ± 2% for pH 2 extracted samples were lower than expected. However, compared to the extraction efficiency for marine DOC of 42 ± 7% (n = 187) found in another study [55], our values are still in the range of uncertainty. Possible reasons for the lower DOC extraction efficiencies might be (i) unknown influence of the source material (ii) too high original DOC measurements or (iii) a problem with the adsorber material. Nevertheless, the results of the quadruplicates of each treatment in our extractions were very consistent, reproducible and invariant, ensuring comparability of our samples.

It has been previously shown that changes in salinity and DOM quality (e.g. at different sample locations in estuaries) can affect DOM recovery via SPE [92, 93]. However, we found no significant effect of salinity on the amount of recovered DOC. Similar DOC extraction efficiencies throughout different salinities suggest little or no fractionation effects as a result of changes in DOM quality. These findings are further supported by an additional experiment, in which low salinity samples from the Weser River were spiked with different concentrations of NaCl and extracted using PPL cartridges (S1 File). The results also showed that the DOC extraction efficiency was not affected by salinity. However, structural changes (indicated by changes in polarity) were observed with changes in salinity, similar to our samples. Thus, we conclude that the polarity of some organic compounds can be reduced by the presence of salt. Structural changes with changes in ionic strength of the medium were also observed for humic acids [94].

Acidification of samples prior to SPE yielded significantly higher DOM_SPE_ and metal-DOM_SPE_ concentrations in the methanol extracts compared with samples extracted at neutral pH and thus gave a more comprehensive picture to discuss changes in DOM stoichiometry and polarity characteristics with changing salinity. It has been shown that acidification leads to higher extraction efficiencies for natural organic matter due to the protonation of functional groups such as organic acids and phenols [38]. Overall, our method is only suitable to extract specific fractions of the natural metal-organic complex pool: the strong acidic fraction in pH 2 extracted samples and the neutral/weak acidic fraction in pH 8 extracted samples, respectively (as defined by Waska et al.) [79]. Cu-DOM_SPE_ extraction efficiencies were similar to those previously reported for acidified and non-acidified PPL extracts [79]. Mills et al. reported decreasing extraction efficiencies of Cu-organic complexes with decreasing pH and mentioned that acidification to pH ≤ 4 did not allow the existence of stabile Cu-DOM_SPE_ complexes [95]. However, another study indicates that also acid-stable Cu-containing compounds can occur in natural aquatic environments [96]. This contradiction reflects that the stability of Cu-organic complexes also depends on the acid-base characteristics of the Cu-binding functional groups and their competitive binding with H^+^ and possibly other major ions such as Ca^2+^ and Mg^2+^. In summary, acidification leads to two competing effects on the recovery of DOM_SPE_ and metal-DOM_SPE_: (i) an increase in the carbon extraction efficiency due to the protonation of organic matter and (ii) a decrease in the complex extraction efficiency due to reduced stability of protonated/acidified organic complexes. For the riverine samples E1 and W1, acidification led to a relative increase by factor 3–4 in both DOC_SPE_ and DOS_SPE_ concentrations, respectively, compared to the non-acidified samples. For trace metals, the net effect of both processes is reflected in differences in average molar DOC_SPE_/metal-DOM_SPE_ ratios of pH 2 and pH 8 extracted samples, respectively, which were about a factor of 1.5–2 for Cu and Ni while no net effect was observed for Co. For estuarine and marine samples however, the decrease in trace metals changed by factor 1.3 – 1.7 for Co and 3 – 4 for Ni and Cu, whereas the increase in DOC_SPE_ and DOS_SPE_ concentrations remained similar to those found in riverine samples. Thus, we can conclude that acidification prior to SPE plays an important role, since it improves the recovery of both DOM_SPE_ and metal-DOM_SPE_. As a result, we found higher extraction efficiencies for pH 2 extracted metal-DOM_SPE_ compared to pH 8 as similarly found for DOM_SPE_. However, acidification likely leads to changes in the quality of organic ligands. In contrast to DOC and Co, we found a significant correlation of the Cu extraction efficiency with salinity. This can have several reasons: (i) differences in the quality of riverine and marine organic ligands (e.g. a higher binding strength of marine Co-complexing ligands) as indicated by differences in polarity or (ii) ionic strength of the medium. The first assumption is supported by a higher polarity of terrestrial DOM which goes along with lower DOC_SPE_/metal-DOM_SPE_ ratios. The second assumption might be explained by an increasing amount of inorganic ions with increasing ionic strength of the medium that could compete with the trace metal ions.

## Conclusion

In this study we presented the concentration and distribution of DOC_SPE_, DON_SPE_, DOS_SPE_, and dissolved metal-DOM_SPE_ with changing salinity in two rivers draining to the North Sea. With regard to the research question/hypothesis stated in the introduction, we can conclude:

- DOM_SPE_ concentrations decreased from riverine to marine waters. The differences in the relative changes in DOC_SPE_ and DOS_SPE_ concentration suggest a preferential removal of DOS_SPE_ over DOC_SPE_ (and DON_SPE_).
- The concentration of some solid-phase extractable trace metals (^52^Cr, ^59^Co, ^60^Ni, ^63^Cu) was correlated with the DOC_SPE_ and DOS_SPE_ concentrations as a result of the presence of organic complexes. The positive correlations of the DOC_SPE_/Co-DOM_SPE_ and DOC_SPE_/Ni-DOM_SPE_ ratios with the DOC_SPE_/DOS_SPE_ ratio and relatively high extraction efficiencies for Co and Cu suggest complexation of trace metals with organic carbon- and sulfur-containing ligands. Increasing Co and Cu concentrations after UV digestion further supported the presence of strong organic sulfur-trace metal complexes. The affinity of trace metals to (sulfur-containing) organic ligands followed the Irving-Williams order.
- DOM polarity reflected typical changes along the estuary from highly polar terrestrial DOM in riverine waters to non-polar DOM compounds in the marine water. This is reflected in a decreasing DOC_pol_/DOC_non-pol_ ratio with increasing salinity.
- Acidification prior SPE plays an important role and leads to a higher recovery of both DOM_SPE_ and metal-DOM_SPE_ compared to neutral SPE. Higher DOC yield by acidification is more important for the metal yield than the negative effect of acidification on complexation. On a qualitative scale, however, acidification can of course make a big difference for the recovery of different organic ligands.

## Supporting information

S1 Fig UV peak area at 210 nm versus DOC_SPE_ concentrations of all samples. A significant linear correlation was found for both fractions: the low concentrated fraction (all pH 8 extracted samples and the pH 2 extracted marine sample) with DOC_SPE_ concentrations from 0–40 μmol L^-1^ (unfilled symbols) and the high concentrated fractions (pH 2 extracted riverine and estuarine samples) with DOC_SPE_ concentrations > 100 μmol L^-1^ (filled symbols).
(DOCX)

S1 Table Limits of detection for all elements analyzed by ICP-MS, given that solid-phase extraction was performed with an enrichment factor of 430. These values were calculated according to DIN 32645.
(DOCX)

S1 File This file includes methodical information about the salt-spiking experiment of riverine samples. (DOCX)
