# Supplementary material for: Stoichiometry, polarity, and organometallics in solid-phase extracted dissolved organic matter of the Elbe-Weser estuary
Source: PLoS One. 2018 Sep 5;13(9):e0203260. doi: 10.1371/journal.pone.0203260 (PMC6124745; doi:10.1371/journal.pone.0203260)
Supplement: S1 Fig — A significant linear correlation was found for both fractions: the low concentrated fraction (all pH 8 extracted samples and the pH 2 extracted marine sample) with DOCSPE concentrations from 0–40 μmol L-1 (unfilled symbols) and the high concentrated fractions (pH 2 extracted riverine and estuarine samples) with DOCSPE concentrations > 100 μmol L-1 (filled symbols). (DOCX) [file pone.0203260.s001.docx]

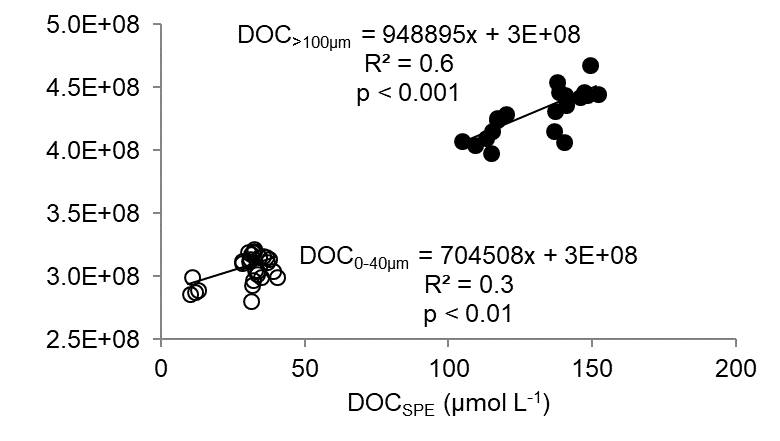


S1 Fig. UV peak area at 210 nm versus DOC_SPE_ concentrations of all samples. A significant linear correlation was found for both fractions: the low concentrated fraction (all pH 8 extracted samples and the pH 2 extracted marine sample) with DOC_SPE_ concentrations from 0 – 40 µmol L^-1^ (unfilled symbols) and the high concentrated fractions (pH 2 extracted riverine and estuarine samples) with DOC_SPE_ concentrations > 100 µmol L^-1^ (filled symbols).
