# Supplementary material for: Stoichiometry, polarity, and organometallics in solid-phase extracted dissolved organic matter of the Elbe-Weser estuary
Source: PLoS One. 2018 Sep 5;13(9):e0203260. doi: 10.1371/journal.pone.0203260 (PMC6124745; doi:10.1371/journal.pone.0203260)
Supplement: S1 Table — These values were calculated according to DIN 32645. (DOCX) [file pone.0203260.s002.docx]

**S1 Table. Limits of detection for all elements analyzed by ICP-MS, given that solid-phase extraction was performed with an enrichment factor of 430.** These values were calculated according to DIN 32645.

| Isotope | Limit of detection |
| --- | --- |
| ^31^P | 3.402 nmol L^-1^ |
| ^32^S | 0.044 µmol L^-1^ |
| ^51^V | 0.013 nmol L^-1^ |
| ^52^Cr | 0.042 nmol L^-1^ |
| ^55^Mn | 0.027 nmol L^-1^ |
| ^59^Co | 6.27*10^-3^ nmol L^-1^ |
| ^60^Ni | 0.050 nmol L^-1^ |
| ^63^Cu | 0.342 nmol L^-1^ |
| ^75^As | 0.334 nmol L^-1^ |
