# Supplementary material for: Stoichiometry, polarity, and organometallics in solid-phase extracted dissolved organic matter of the Elbe-Weser estuary
Source: PLoS One. 2018 Sep 5;13(9):e0203260. doi: 10.1371/journal.pone.0203260 (PMC6124745; doi:10.1371/journal.pone.0203260)
Supplement: S1 File — (DOCX) [file pone.0203260.s003.docx]

Supporting Information 1 for:

**The interrelation of dissolved organic matter stoichiometry, organic sulfur concentration and trace element complexation along the Elbe-Weser estuary**

Kerstin B. Ksionzek, Jing Zhang, Kai-Uwe Ludwichowski, Dorothee Wilhelms-Dick, Scarlett Trimborn, Thomas Jendrossek, Gerhard Kattner, Boris P. Koch

This file includes methodical information about the salt-spiking experiment of riverine samples.

To estimate the influence of salt on the extraction efficiency of DOM, low salinity Weser River samples (Fig. 1) were taken, and filtered using a stainless steel in-line filter holder (Sartorius type 16275), a peristaltic pump, a pre-filter (GF/F. 13400-142-K. Sartorius) and a membrane filter (cellulose acetate. 0.2 µm pore size. 11107-142-G. Sartorius). The first 500 mL of every filtration were rejected. Samples were stored in the dark at 4°C until further analysis. For the spiking-experiment, the samples were mixed with defined amounts of pre-combusted sodium chloride (NaCl, 500 °C. 5 h). The amount of added NaCl increased continuously in five steps (0, 8.75. 17.5, 26.25, 35 g/L). The final salinities of the sample R1/R2 are given in Table 1. 150 mL of each sample was acidified to pH 2, extracted (PPL, 200 mg. Mega Bond Elut, Agilent Technologies) and eluted with 1.5 mL of methanol (LiChrosolv. Merck) into pre-combusted glass vials. DOC and DOC_SPE_ concentrations were determined by high temperature catalytic oxidation. DOC in original water samples were measured directly. For analysis of DOC_SPE_, 50 µL of the extract were evaporated under N_2_ and subsequently redissolved in 7 mL ultrapure water.

Final DOC and DOC_SPE_ concentrations and according extraction efficiencies are given in Table 1.

**Fig 1. Sampling location of the Weser River samples R1 and R2 (blue dot).** Color represents the surface salinity. Black dots represent stations, at which temperature and salinity were measured. Red dots represent stations, at which samples for DOM and trace element analysis were taken.

**Table 1. DOC and DOC_SPE_ concentrations and according extraction efficiencies of the sodium chloride spiked Weser riverine samples R1 and R2.**

| Sample | DOC  (µmol L^-1^) | DOC_SPE_  (µmol L^-1^) | Salinity | Extraction efficiency (%) |
| --- | --- | --- | --- | --- |
| R1 | 247 | 138 | 0.9 | 56 |
|  |  | 136 | 9.5 | 55 |
|  |  | 137 | 18.1 | 55 |
|  |  | 129 | 26.7 | 52 |
|  |  | 128 | 35.3 | 51 |
|  |  |  |  |  |
| R2 | 201 | 116 | 0.8 | 58 |
|  |  | 112 | 9.4 | 56 |
|  |  | 113 | 18.0 | 56 |
|  |  | 115 | 26.6 | 57 |
|  |  | 118 | 35.2 | 59 |
